# The prevalence of *Escherichia coli* strains with extended spectrum beta-lactamases isolated in China

**DOI:** 10.3389/fmicb.2015.00335

**Published:** 2015-04-21

**Authors:** Haihong Liu, Yueling Wang, Gang Wang, Quantai Xing, Lihua Shao, Xiaomeng Dong, Lintao Sai, Yongjuan Liu, Lixian Ma

**Affiliations:** ^1^Department of Infectious Diseases, Qilu Hospital, Shandong University, Jinan, China; ^2^Department of Clinical laboratory, Shandong Provincial Hospital Affiliated to Shandong University, Jinan, China; ^3^Department of Laboratory Sciences, School of Public Health, Shandong University, Jinan, China

**Keywords:** extended-spectrum-lactamase, *Escherichia coli*, multi-drug resistant, PCR, TEM, CTX-M

## Abstract

The extended-spectrum-lactamases-producing Escherichia coli has rapidly spread worldwide. Escherichia coli has been becoming much more resistant to β-lactam antibiotics and other commonly available antimicrobials. We investigated the prevalence, resistance, and probable gene type of extended spectrum beta-lactamases (ESBLs) using minimum inhibitory concentrations (MICs) testing and polymerase chain reaction (PCR). We have collected 289 single-patient E. coli Isolates based on samples of China from July 2013 to August 2014. This article explored that the prevalence of ESBL-producing Isolates showed multi-resistant to antimicrobials such as fluoroquinolones, trimethoprim, tetracycline and aminoglycosides, and so on. The frequencies of resistance in Isolates were as follows: Ciprofloxacin, 74%, gentamicin, 69.5%, levofloxacin, 63%, tobramycin, 39%, and minocycline, 7.9%. According to our results, 197(68.2%) of the total 289 Isolates were ESBL-producing strains; further, 172 (87.3%) producers contained genes encoding CTX-M enzymes and 142(72.1%) producers contained genes encoding TEM enzymes. Most ESBL-producing Escherichia coli has produced more than one type of β-lactamase. Nucleotide sequence analysis has revealed the diversity of ESBLs types: CTX-M -15 is in the majority and TEM-135, CTX-M-3, CTX-M-98, CTX-M-14, CTX-M-142, CTX-M-65, CTX-M-55, CTX-M-27, and CTX-M-123 have been recovered. The results confirm that ESBL producers which are common in hospital strains of Escherichia coli are resistant to cephalosporins and other antibiotics in China. It is important to monitor such strains closely and provide scientific evidence of rational application of antibiotics to prevent their spread.

## Introduction

The resistance of ESBL-producing *Enterobacteriaceae* has currently become one of the most important nosocomial resistance problems around the world and it has been found that there are much more community-acquired ones (Bradford, 2001; Valverde et al., 2004). *Escherichia coli* has been a universal commensal bacterium causing infections in humans and animals and often leads to urinary tract infections (UTI) and bacteremia for humans (Paterson and Bonomo, 2005). ESBL-producing *Enterobacteriaceae* were firstly reported in the middle of 1980s in Germany, since then a steady increase of these strains have been reported worldwide (Bradford, 2001).

Recently, researchers have paid much more attention to extended spectrum beta-lactamases (ESBLs). As well known, the production of β-lactamase is the most common mechanism of bacterial resistance to β-lactam antibiotics; with the introduction of extended-spectrum cephalosporins, ESBLs has spread across the world (Medeiros, 1997; Yagi et al., 2000; Saladin et al., 2002; Yong and Toleman, 2009). ESBLs could inactivate oxyimino-β-lactams like third-generation cephalosporins and aztreonam, but not hydrolyze the carbapenems, which are highly susceptible to inhibition by clavulanic acid and tazobactam (Livermore, 1995). ESBL-producing strains are not only highly resistant to β-lactam antibiotics but also resistant to aminoglycosides, quinolones, sulfonamides, and so on (Ho et al., 2007).

The epidemic situation, resistance and genotype distribution of ESBL-producing strains have varied with different countries, regions and even hospitals. ESBL strains have extended all over the world stage by stage, while detectable rates have shown significant difference in different hospitals or areas of the same country. Previous researches have indicated the detectable rate of ESBL-producing strains from 5 to 8% of *Escherichia coli* Isolates in Korea, Japan, Malaysia, and Singapore, from 12 to 24% in Thailand, Taiwan, Philippines, and Indonesia (Paterson and Bonomo, 2005), from 62 to 100% in India (Mathai et al., 2009). By contrast, the rate was almost up to 60% in China (Xiao et al., 2011).

Much more genetic subtypes of ESBLs have been found since 1980s, which has exceeded 200 so far. The vast majority of ESBLs belong to three types, including TEM, SHV, and CTX-M types (Bauernfeind et al., 1990; Barthelemy et al., 1992; Bradford, 2001; Paterson and Bonomo, 2005; Livermore et al., 2007; Cantón et al., 2012). Previous researches exploring TEM-type has mainly involved the predominant genotype (particularly, TEM-10, TEM-12, and TEM-26) in north America (Rasmussen et al., 1993; Urban et al., 1994); 92% ESBL-positive *E. coli* Isolates expressed a CTX-M-type enzyme from 2001 to 2006 and CTX-M-1 being the predominant group in Sweden (Fang et al., 2008); the CTX-M-15 enzyme was the most prevalent enzyme in United Kingdom (Baraniak et al., 2005; Livermore et al., 2007). In China, the CTX-M β-lactamases was the most prevalent ESBLs, mainly including CTX-M-3, –9, –14, and –15 (Xiong et al., 2002; Yu et al., 2007; Liu et al., 2009). In short, there exists tremendous variability in antimicrobial resistance amongst pathogens in different regions or in different hospitals located the same regions.

Given few wide-ranging survey researches in Shandong province, China, we aimed to explore ESBLs epidemiology, resistance and resistant genotypes of 289 *E. coli* strains Isolated from three hospitals in that area. In addition, we have collected clinical data about the patients to carry on further research. The study helps us reveal their distributions and local constitutions in that area, thus providing guidance for clinical treatment, and then contributing to avoiding delay the conditions of patients and medical resources waste.

## Materials and Methods

### Bacterial Strains

We have collected 289 clinical Isolates of *E. coli* from patients in three typical hospitals of Shandong province in, China, from July 2013 to August 2014. There exist no replicate strains Isolated from one patient in our samples. Additional information has been collected with each Isolate, including isolation date, origin of the specimen, demographic details. Our experiment has been approved by Medical Ethics Committee of Shandong University School of Medicine (Grant No. 201401048).

### ESBL Identification and Antimicrobial Susceptibility Testing

Initially, all Isolates were tested using the Kirby Bauer disk diffusion method according to the Clinical Laboratory Standards Institute (2013). *E. coli* ATCC25922 was used as control strains. The tested antibiotics included: ampicillin, ampicillin/sulbactam, amoxicillin/clavulanate, ticarcillin, piperacillin, piperacillin/tazobactam, cephalothin, cefuroxime, cefoxitin, ceftriaxone, cefotaxime, ceftazidime, cefepime, meropenem, and aztreonam (Mast Diagnostics, Merseyside, UK). co-trimoxazole, gentamicin, nalidixic acid, ciprofloxacin, imipenem, meropenem, tetracycline, and nitrofurantoin (Oxoid Ltd., Basingstoke, UK). MIC values were extrapolated by the BIOMIC automated reading system and software package (Giles Scientific, New York, NY, USA).

The disk diffusion method was applied to assess ESBL production in all the Isolates with cefotaxime (30 ug) and ceftazidime (30 ug) alone and in combination with clavulanic acid (10 ug) as recommended by the Clinical and Laboratory Standards Institute (CLSI).

### Nucleic Acid Extractions

DNA had been extracted from the Isolates by using boiling methods. Each strain was suspended in 500 ul of distilled water at concentration of MacFarland 0.5 and boiled at 100°C for 10 min. Then spined it in a centrifuge and kept the supernatant fluid in the –20°C freezer.

### Polymerase Chain Reaction Detection and Sequencing of ESBLs

To determine the genotype of ESBLs, we performed polymerase chain reaction (PCR) amplification with the TEM and CTX-M. ESBLs Isolates were amplified with primers (Essack et al., 2001): 5′-ATGAGTATTC AACATTTCCGTG-3 and 5′-TTACCAATGCTTAATCAGTGAG-3, which have been designed to amplify TEM gene (length, 840 bp). Cycling conditions were as following: initial denaturation at 95°C for 3 min; 35 cycles of 95°C for 1 min, 55°C for 1 min and 72°C for 1 min; and a final elongation at 72°C for 5 min. We applied CTX-M gene primers: C1 (5′-SCSATGTGCAGYACCAGTAA-3) and C2 (5′-CCGCRAT ATGRTTGGTGGTG-3) to detect CTX-M gene (length, 554 bp). Cycling conditions were as follows: initial denaturation at 94°C for 3 min; 35 cycles of 94°C for 30S, 55°C for 30s and 72°C for 45s; and a final elongation at 72°C for 5 min. PCR reactions for each strain were repeated at least three times.

### Sequencing of TEM and CTX-M genes

The PCR products were purified with a QIA quick PCR purification kit (Qiagen, Hilden, Germany) and sequenced on an ABI Prism 377 automated sequencer (Perkin-Elmer, Norwalk, CT, USA). The primers for sequencing were the amplification primer. All sequences were confirmed by two independent determinations and analyzed by Basic Local Alignment Search Tool.

## Results

### Bacterial Strains

In our samples, these strains were Isolated mainly from patients in urinary surgery ward 110 (38.1%) and ICU 63 (21.8%). The rest in turn were derived from Respiratory Medicine, 27 (9.3%), Neurosurgery 22 (7.6%), Thoracic Surgery and General Surgery 21 (7.3%), Oncology 11 (3.8%), Orthopedic Surgery 9 (3.1%), Cardiovascular Medicine 9 (3.1%), Gastroenterology 8 (2.8%), Endocrinology 7 (2.4%), Neurology 2 (0.7%). A total of 197 ESBL-producing Isolates were detected phenotypically using the CLSI criteria for ESBL screening and disk confirmation test, the detection rate being 68.2% (197/289) (See Table 1). We found that much more ESBL-producing Isolates exited in urinary surgery ward and ICU and the detection rate were higher than the other wards. The cohort of 197 patients had a mean age of 53.8, which ranges from 20 to 88 (See Table 2). Of all Isolates, 109 (55.3%) patients were over 60. According to statistical results by age, we concluded that we should further enhance elderly patients’ management and treatment dynamics, completes the prevention and treatment work. The main sources of ESBL- producing *E-coil* were unire 96 (48.73%, of total ESBL-producing Isolates), wounds 27 (13.71%), sputa 26 (13.20%), genital secretion 16 (8.12%), hydrothorax and ascite 15 (7.61%), blood 12 (6.09%), bile 5 (2.54%) (See Table 3).

**TABLE 1:** **Distribution of strains in different wards**.

Wards	Total	ESBLs	Detection rate
Urology	110	74	67.27%
Intensive care unit	64	54	84.38%
Respiratory medicine	27	17	62.96%
Neurosurgery	22	13	59.09%
Thoracic surgery and general surgery	21	13	61.90%
Oncology	11	7	63.64%
Cardiovascular medicine	9	3	33.33%
Orthopedic surgery	9	5	55.56%
Endocrinology	8	5	62.50%
Gastroenterology	6	4	66.67%
Neurology	2	1	50.00%
Total	289	197	68.2%

**TABLE 2:** **Frequency of ESBL Isolates by age**.

Age					
Gender	20–40	40–60	60–80	>80	Total
Male	15	24	43	14	96
Female	21	28	40	12	101
Total	36	52	83	26	197

**TABLE 3:** **Distribution of *E. coli* Isolates by type of specimen**.

Specimen type	Total	ESBLs	Non-ESBLs
Unire	126	96	30
Wounds	42	27	15
Sputa	38	26	12
Genital secretion	28	16	12
Hydrothorax and ascite	26	15	11
Blood	20	12	8
Bile	9	5	4
Total	289	197	92

### Antimicrobial Susceptibility

The ESBL-producing Isolates were often multidrug-resistant. ESBL producers have shown much higher rates of resistance than those non-ESBL to ciprofloxacin (ESBL versus non-ESBL, 74% versus 30%, *P* < 0.01), gentamicin (69.5% versus 28.5%, *P* < 0.01), co-trimoxazole (75.8% versus 36%, *P* < 0.01), aztreonam (79% versus 20.5%, *P* < 0.01), and levofloxacin (63% versus 40%, *P* < 0.01). The great majority of Isolates were susceptible to Minocycline (ESBL versus non-ESBL, 82% versus 92.1%). All Isolates were susceptible to amikacin and imipenem (χ^2^ test for all groups).

### Characteristics of the ESBL-producing Isolates

All 197 Isolates with an ESBL phenotype tested positive for blaCTX-M and blaTEM using the consensus primers. We found 172 (87.3%) Isolates were positive for blaCTX-M, while 142(72.1%) Isolates were positive for blaTEM and 11 non-TEM-CTX-M-type Producers. In addition, most Isolates appeared to produce both TEM and CTX-M enzymes (128). Using nucleotide sequence analysis we found that the TEM enzymes detected two types which were TEM-1(138) and TEM-135(4) β-lactamase. Unlike the TEM enzymes, the CTX-M enzymes with a variety of types included CTX-M-3(16), CTX-M-14(77), CTX-M-15(64), CTX-M-55(9), CTX-M-64(1), CTX-M-65(2) and CTX-M-98(3) and the genes of CTX-M-14 and CTX-M-15 were in the majority.

## Discussion

In the article, 289 *E. coli* Isolates were collected from patients from July 2013 to August 2014; the detection rate of ESBL producers was 68.2% which was significantly higher than previous researches (Xiao et al., 2011). Therefore, we should pay more attention to the problem and try to take appropriate prevention and remedial measures. We found that much more ESBL-producing Isolates in urinary surgery ward and ICU and the detection rate were higher than the other wards. Because a majority of patients in ICU acquired serious diseases lowering their immunity, and involved heavy use of antibiotics for a long time, both of which contributing to higher detection rates. And for patients in urinary surgery, higher detection rates were related to their selves characteristics, one of which was mainly urinary tract obstruction in favors of bacteria propagation, in addition to urethral catheterization, further increasing opportunities for infection. Besides, we found most ESBL-producing strains occur in old man or woman, because the elderly themselves have low immunity. And in the patients, one patient over 60 repeatedly infected with the ESBL-producing *E. coli*. As a long duration of extended-spectrum β-lactamase-producing *Enterobacteriaceae* carriage after discharge (Birgand et al., 2013), we concluded that those patients underlying chronic illnesses which had lowered their immunity were apt to be infected with ESBL-producing *E. coli*. Therefore, we should initiate active interventions and treatments.

The susceptibility test data showed that the ESBL producers which were resistant to most-lactams and frequently resistant to the non-β-lactam antibiotics, for example, fluoroquinolones and aminoglycosides. If patients are infected by ESBL producers, cefmetazole, or imipenem will be chosen before results of antibiotic susceptibility test having been done. But if patients are in a critical condition, we should make choice of carbapenems (Paterson, 2000). ESBL producers have shown much higher rates of resistance than those non-ESBL. Therefore, we should avoid antibiotics abuse in outpatient clinic and community hospitals, reducing opportunities for emergence of ESBLs.

TEM is the main type of β-lactamase, the TEM-1 group being the most common ones. CTX-M enzymes was a new group of plasmid-mediated ESBLs which has become the predominant ESBLs reported in Europe from last decade and has increased dramatically in many countries (Bonnet, 2004; Cantón and Coque, 2006; Livermore et al., 2007; Pitout and Laupland, 2008). In different geographic areas, the antibiotic consumption and dissimilar risk factors might have also contributed to the current epidemiology of CTX-M enzymes. Our study found that the CTX-M-14 and CTX-M-15 were in the majority, Dolejska et al. (2011) and Hiroi et al. (2012) coincided with that.

Even though we have explored ESBLs epidemiology, resistance and resistant genotypes in three hospitals of Shandong province, with limitation of small samples, we cannot provide enough theoretical and practical guidance for clinical treatment. In the future research, we should base upon more large survey samples from perspective of increasing patients, hospitals and regions. On the other hand, because of much more ESBL producers resistant to carbapenems, to conduct a targeted research, we will focus on carbapenem-resistant *Enterobacteriaceae* (Centers for Disease Control and Prevention, 2013; Asakura et al., 2014). Given carbapenems being the first-line agent for patients with ESBL-producing bacteria, the occurrence of resistant organisms could be related to carbapenems, and then we will explore whether non-carbapenems plays more efficient role in infections with ESBL-producing *Enterobacteriaceae*.

Generally speaking, much more ESBL producers are detected among *E. coli* in China. The drug resistance rate of *E. coli* is high, with the tendency getting higher. Attaching more importance to monitor the drug resistance of bacterium and reasonable application and control abuse of antibiotics in clinic. Based on local database, our research have clarified the clinical and epidemiological features in Shandong province, which contributes to provide guidance for identifying appropriate treatment plans according to patients characteristics, especially for patients underlying chronic illnesses. We should take measures appropriately, and then contributing to avoiding delay the conditions of patients and medical resources waste.

### Conflict of Interest Statement

The authors declare that the research was conducted in the absence of any commercial or financial relationships that could be construed as a potential conflict of interest.

## References

[B1] Asakura T. Ikeda M. Nakamura A. Kodera S. (2014). Efficacy of empirical therapy with non-carbapenems for urinary tract infections with extended-spectrum β-lactamase- producing *Enterobacteriaceae*. Int. J. Infect. Dis. 29, 91–95. 10.1016/j.ijid.2014.08.018 25461239

[B2] Baraniak A. Fiett J. Mrówka A. Walory J. Hryniewicz W. Gniadkowski M. (2005). Evolution of TEM-type extended-spectrum β-lactamases in clinical *Enterobacteriaceae* strains in Poland. Antimicrob. Agents Chemother. 49, 1872–1880. 10.1128/AAC.49.5.1872-1880.2005 15855509 PMC1087658

[B3] Barthelemy M. Peduzzi J. Bernard H. Tancrede C. Labia R. (1992). Close amino-acid sequence relationship between the new plasmid-mediated extended-spectrum L-lactamase MEN-1 and chromosomally encoded enzymes of *Klebsiella oxytoca*. Biochim. Biophys. Acta 1122, 15–22. 10.1016/0167-4838(92)90121-S 1633193

[B4] Bauernfeind A. Grimm H. Schweighart S. (1990). A new plasmidic cefotaximase in a clinical Isolate of *Escherichia coli*. Infection 18, 294–298. 10.1007/BF01647010 2276823

[B5] Birgand G. Armand-Lefevre L. Lolom I. Ruppe E. Andremont A. Lucet J. C. (2013). Duration of colonization by extended-spectrum β-lactamase- producing *Enterobacteriaceae* after hospital discharge. Am. J. Infect. Control. 41, 443–447. 10.1016/j.ajic.2012.05.015 22998785

[B6] Bonnet R. (2004). Growing group of extended-spectrumβ-lactamases: the CTX-M enzymes. Antimicrob. Agents Chemother. 48, 1–14. 10.1128/AAC.48.1.1-14.2004 14693512 PMC310187

[B7] Bradford P. A. (2001). Extended-spectrum β-lactamases in the Twenty-first century: characterization, epidemiology, and detection of this important resistance treat. Clin. Microbiol. Rev. 14, 933–951. 10.1128/CMR.14.4.933-951.2001 11585791 PMC89009

[B8] Cantón R. Coque T. M. (2006). The CTX-M β-lactamase pandemic. Curr. Opin. Microbiol. 9, 466–475. 10.1016/j.mib.2006.08.011 16942899

[B9] Cantón R. González-Alba J. M. Galán J. C. (2012). CTX-M enzymes: origin and diffusion. Front. Microbiol. 26:1–19. 10.3389/fmicb.2012.00110 22485109 PMC3316993

[B10] Centers for Disease Control and Prevention. (2013). Vital signs: carbapenemQ11 resistant, Enterobacteriaceae. Morb. Mortal. Wkly. Rep. 62, 165–170. 23466435 PMC4604788

[B11] Clinical Laboratory Standards Institute. (2013). Performance Standards for Antimicrobial Susceptibility Testing; Twenty-Third Informational Supplement M100-S23, Vol. 33, Wayne, PA: National Committee for Clinical Laboratory Standards.

[B12] Dolejska M. Frolkova P. Florek M. Jamborova I. Purgertova M. Kutilova I. (2011). CTX-M-15-producing *Escherichia coli* clone B2-O25b-ST131 and *Klebsiella* spp. Isolates in municipal wastewater treatment plant effluents. J. Antimicrob. Chemother. 66, 2784–2790. 10.1093/jac/dkr363 21954457

[B13] Essack S. Y. Hall L. M. Pillay D. G. McFadyen M. L. Livermore D. M. (2001). Complexity and diversity of *Klebsiella pneumoniae* strains with extended-spectrum β-lactamases isolated in 1994 and 1996 at a teaching hospital in Durban, South Africa. Antimicrob. Agents Chemother. 45, 88–95. 10.1128/AAC.45.1.88-95.2001 11120950 PMC90245

[B14] Fang H. Ataker F. Hedin G. Dornbusch K. (2008). Molecular epidemiology of extended-spectrum β-lactamases among *Escherichia coli* Isolates collected in a Swedish hospital and its associated health care facilities from 2001 to 2006. J. Clin. Microbiol. 46, 707–712. 10.1128/JCM.01943-07 18094139 PMC2238137

[B15] Hiroi M. Yamazaki F. Harada T. Takahashi N. Iida N. Noda Y. (2012). Prevalence of extended-spectrum β-lactamase-producing *Escherichia coli* and *Klebsiella pneumoniae* in food-producing animals. J. Vet. Med. Sci. 74, 189. 10.1292/jvms.11-0372 21979457

[B16] Ho P. L. Poon W. W. Loke S. L. Leung M. S. Chow K. H. Wong R. C. (2007). Community emergence of CTX-M type extended-spectrum β-lactamases among urinary *Escherichia coli* from women. J. Antimicrob. Chemother. 60, 140–144. 10.1093/jac/dkm144 17496058

[B17] Liu W. Chen L. Li H. Duan H. Zhang Y. Liang X. (2009). Novel CTX-M β-lactamase genotype distribution and spread into multiple species of *Enterobacteriaceae* in Changsha, southern China. J. Antimicrob. Chemother. 63, 895–900. 10.1093/jac/dkp068 19297379

[B18] Livermore D. M. (1995). β-Lactamases in laboratory and clinical resistance. Clin. Microbiol. Rev. 8, 557–584. 8665470 10.1128/cmr.8.4.557 PMC172876

[B19] Livermore D. M. Canton R. Gniadkowski M. Nordmann P. Rossolini G. M. Arlet G. (2007). CTX-M: changing the face of ESBLs in Europe. J. Antimicrob. Chemother. 59, 165–174. 10.1093/jac/dkl483 17158117

[B20] Mathai D. Manoharan A. Vasanthan G. (2009). “Epidemiology and implications of ESBL,” in 1st Edn, Vol. 14, Criticai Care Update, eds Nayyar V. Peter J. V. Kishan R. Srinivas S. (New Delhi: Jaypee Brothers Medical Publishers (P) Ltd.), 152–162.

[B21] Medeiros A. A. (1997). Evolution and dissemination of β-lactamases accelerated by generations of β-lactam antibiotics. Clin. Infect. Dis. 24(Suppl. 1), S19–S45. 10.1093/clinids/24.Supplement_1.S19 8994778

[B22] Paterson D. L. (2000). Recommendation for treatment for treatment of severe infections caused by *Enterobacteriaceae* producing extended spectrum β-lactamases (ESBLs). Clin. Microbiol. Infec. 6, 460–463. 10.1046/j.1469-0691.2000.00107.x 11168179

[B23] Paterson D. L. Bonomo R. A. (2005). Extended-spectrum βlactamases: a clinical update. Clin. Microbiol. Rev. 18, 657–686. 10.1128/CMR.18.4.657-686.2005 16223952 PMC1265908

[B24] Pitout J. D. D. Laupland K. B. (2008). Extended-spectrumβ-lactamase-producing *Enterobacteriaceae*: an emerging public-health concern. Lancet Infect. Dis. 8, 159–166. 10.1016/S1473-3099(08)70041-0 18291338

[B25] Rasmussen B. A. Bradford P. A. Quinn J. P. Wiener J. Weinstein R. A. Bush K. (1993). Genetically diverse ceftazidime-resistant Isolates from a single center: biochemical and genetic characterization of TEM-10 β-lactamases encoded by different nucleotide sequences. Antimicrob. Agents Chemother. 37, 1989–1992. 10.1128/AAC.37.9.1989 8239618 PMC188106

[B26] Saladin M. Cao V. T. Lambert T. (2002). Diversity of CTX-M β-lactamases and their promoter regions from *Enterobacteriaceae* Isolated in three Parisian hospitals. FEMS Microbiol. Lett. 209, 161–168. 10.1016/S0378-1097(02)00484-6 12007800

[B27] Urban C. Meyer K. S. Mariano N. Rahal J. J. Flamm R. Rasmussen B. A. (1994). Identification of TEM-26 β-lactamase responsible for a major outbreak of ceftazidime-resistant *Klebsiella pneumoniae*. Antimicrob. Agents Chemother. 38, 392–395. 10.1128/AAC.38.2.392 8192474 PMC284466

[B28] Valverde A. Coque T. M. Sánchez-Moreno M. P. Rollán A. Baquero F. Cantón R. (2004). Dramatic increase in prevalence of fecal carriage of extended-spectrum β-lactamase-producing *Enterobacteriaceae* during nonoutbreak situations in Spain. J. Clin. Microbiol. 42, 4769–4775. 10.1128/JCM.42.10.4769-4775.2004 15472339 PMC522353

[B29] Xiao Y. H. Giske C. G. Wei Z. Q. Shen P. Heddini A. Li L. J. (2011). Epidemiology and characteristics of antimicrobial resistance in China. Drug Resist. Updat. 14, 236–250. 10.1016/j.drup.2011.07.001 21807550

[B30] Xiong Z. Zhu D. Wang F. Zhang Y. Okamoto R. Inoue M. (2002). Investigation of extended-spectrum-lactamase in *Klebsiella pneumonia* and *Escherichia coli* from China. Diagn. Micr. Infec. Dis. 44, 195–200. 10.1016/S0732-8893(02)00441-8 12458128

[B31] Yagi T. Kurokawa H. Shibata N. Shibayama K. Arakawa Y. (2000). A preliminary survey of extended-spectrum L-lactamases (ESBLs) in clinical Isolates of *Klebsiella pneumoniae* and *Escherichia coli* in Japan. FEMS Microbiol. Lett. 184, 53–56. 10.1016/S0378-1097(00)00016-1 10689165

[B32] Yong D. Toleman M. A. (2009). Characterization of a new metallo-β-lactamase gene, bla_NDM–1_, and a novel erythromycin esterase gene carried on a unique genetic structure in *Klebsiella pneumoniae* sequence type 14 from India. Antimicrob. Agents Chemother. 53, 5046–5054. 10.1128/AAC.00774-09 19770275 PMC2786356

[B33] Yu Y. Ji S. Chen Y. Zhou W. Wei Z. Li L. (2007). Resistance of strains producing extended-spectrum β-lactamases and genotype distribution in China. J. Infect. 54, 53–57. 10.1016/j.jinf.2006.01.014 16533535

